# Understanding Sociodemographic Factors and Reasons Associated with COVID-19 Vaccination Hesitance among Adults in Tanzania: A Mixed-Method Approach

**DOI:** 10.4269/ajtmh.23-0229

**Published:** 2023-09-11

**Authors:** Hajirani M. Msuya, Gumi A. Mrisho, Abdallah Mkopi, Mwifadhi Mrisho, Omar N. Lweno, Ali M. Ali, Ali H. Said, Michael G. Mihayo, Sara S. Mswata, Anneth M. Tumbo, Grace Mhalu, Said A. Jongo, Kamaka R. Kassim, Gloria D. Nyaulingo, Silas G. Temu, Paul E. Kazyoba, Hussein Haruna, Rogath Kishimba, Hellen Kassa, Grace W. Mwangoka, Salim Abdulla

**Affiliations:** ^1^ Ifakara Health Institute, Dar es Salaam, Tanzania;; ^2^ National Institute for Medical Research, Dar es Salaam, Tanzania;; ^3^ Ministry of Health, Dodoma, Tanzania;; ^4^ Foundation for Innovation and New Diagnostics, Geneva, Switzerland

## Abstract

Although studies on COVID-19 vaccine hesitancy are being undertaken widely worldwide, there is limited evidence in Tanzania. This study aims to assess the sociodemographic factors associated with COVID-19 vaccine hesitancy and the reasons given by unvaccinated study participants. We conducted a mixed-method cross-sectional study with two components—health facilities and communities—between March and September 2022. A structured questionnaire and in-depth interviews were used to collect quantitative and qualitative data, respectively. A total of 1,508 individuals agreed to participate in the survey and explained why they had not vaccinated against COVID-19. Of these participants, 62% indicated they would accept the vaccine, whereas 38% expressed skepticism. In a multivariate regression analysis, adult study participants 40 years and older were significantly more likely to report not intending to be vaccinated (adjusted odds ratio [AOR], 1.28; 95% CI, 1.01–1.61; *P* = 0.04) than youth and middle-aged study participants between 18 and 40 years. Furthermore, female study participants had a greater likelihood of not intending to be vaccinated (AOR, 1.51; 95% CI, 1.19–1.90; *P* = 0.001) than male study participants. The study identified fear of safety and short-term side effects, and lack of trust of the COVID-19 vaccine; belief in spiritual or religious views; and belief in local remedies and other precautions or preventive measures as the major contributors to COVID-19 vaccine hesitancy in Tanzania. Further empirical studies are needed to confirm these findings and to understand more fully the reasons for vaccine hesitancy in different demographic groups.

## INTRODUCTION

Globally, more than 6.8 million people died as a result of COVID-19, with more than 755 million confirmed cases as of February 2023. From the cases reported worldwide, the African region had more 9.0 million COVID-19 cases and 175,270 fatalities, with 42,717 cases and 846 fatalities reported in Tanzania.^1^ The common symptoms of infection include fatigue, muscle pain, sneezing, a sore throat, and death.^2^^–^^4^ Cleaning your area, avoiding sneezing and coughing in public places, handwashing with soap and sanitizer, and covering your mouth and nose with a mask while sneezing and coughing are all regular recommendations to reduce COVID-19 infection.^5^

Despite these recommended prevention measures, vaccinations have been recognized as the most effective approach in controlling the spread and severity of COVID-19, and have been used as a public health intervention to halt the transmission and evolution of infectious diseases over time.^6^ The vaccination strategy is implemented by 1) vaccinating directly those who are most vulnerable to severe outcomes and 2) protecting them indirectly by vaccinating those who are prone to transmitting the disease,^7^^,^^8^ thereby making the current COVID-19 vaccines a viable option for reducing morbidity and mortality.^7^^,^^9^ However, COVID-19 vaccination rates have been found to be low in the Middle East, Russia, Africa, and several European countries,^10^^–^^12^ and have been attributed to vaccine hesitancy.

Vaccine hesitancy is defined as a delay in accepting vaccinations, or being reluctant or outright refusing to vaccinate, despite the availability of vaccination services.^13^^,^^14^ Vaccine hesitancy was identified as one of the top 10 threats to global health by the WHO in 2019.^13^ A complex decision-making process that includes communication and media; historical influences; religion, culture, gender, socioeconomic, political, and geographic barriers; vaccination experience; risk perception; and vaccination program design ultimately leads to vaccine hesitancy.^14^ Furthermore, one study^15^ found that COVID-19 vaccine hesitation is associated with female gender, young age, low income, and low education level. In addition, the frequently cited reasons for vaccine hesitancy are perceived risks versus benefits, religious beliefs, and a lack of knowledge and awareness of the COVID-19 vaccine.^16^^,^^17^

To reduce transmission and achieve herd immunity, it is estimated that at least 60% to 70% of the population should be vaccinated.^18^ To achieve this end point, the WHO outlined actions the global community must take to vaccinate 40% of the global population against COVID-19 by the end of 2021, and 70% by June 2022.^19^ More than 11.3 billion doses of the COVID-19 vaccine have been given worldwide in support of the WHO’s call for universal vaccination by June 2022, but only 11% of people in low-income countries have received the vaccine, compared with 73% of people in high-income countries.^20^ The African region received 833.5 million doses and only 18% of the population is fully vaccinated.^20^ In July 2021, the United States sent Tanzania a shipment of 1,058,450 doses of the Johnson & Johnson COVID-19 vaccine, followed by 1,065,600 doses of Sinopharm vaccines from the Chinese government in October 2021.^21^

When the first batch of the COVID-19 vaccine arrived in Tanzania in July 2021,^22^ the government prioritized health-care workers, people with comorbidities, adults older than 50 years, port-of-entry workers, military and security forces, and school teachers^23^ to be vaccinated against COVID-19. Because of the perceived high risk, the Tanzanian government changed the policy in August 2021: everyone 18 years and older could receive the COVID-19 vaccine voluntarily, both on the mainland and on Zanzibar.^24^ According to the WHO, Tanzania administered more than 35.9 million doses of the vaccine by February 20, 2023^25^; however, a recent review found Tanzania to be one of several African countries (e.g., Angola, Chad, Republic of Congo, Mali, Mauritania, and Namibia) that lacked data on COVID-19 vaccine acceptance rates.^26^

Despite numerous studies indicating that vaccination prevents severe illness, hospitalization, and death,^27^ people in many Africans countries still refuse or postpone vaccination.^28^ In Tanzania, little is known about COVID-19 vaccine hesitancy. For example, Masele and Daud^29^ concluded that the extent to which information is channeled through a specific source and how it is manipulated affects vaccine hesitancy. Their study focused solely on information sources, particularly misinformation, as reported by Chilongola et al.,^21^ that failed to link their study findings with the sociodemographic characteristics of their study population. Also, misinformation was reported by Konje et al.,^30^ and they added other reasons such as lacking of reliable information on efficacy and safety. In addition, the same study did not observe whether there was a significance association between vaccine hesitancy and gender. A recent qualitative study of public health officials by Yamanis et al.^31^ did not investigate community perceptions that challenged mass COVID-19 vaccination. Another study, by Msuya et al.,^24^ did not include qualitative findings that investigate the barriers and facilitators of COVID-19 vaccination.

Because Tanzania delayed in joining the COVID-19 Vaccines Global Access initiative program after opting for local-context COVID-19 preventive measures, it is critical to understand the drivers of vaccine hesitancy in Tanzania to develop tailored strategies for improving vaccination coverage, increasing acceptability of the COVID-19 vaccine, and decreasing vaccination hesitancy to bring the pandemic to an end quickly. Hence, our study was conducted to access the sociodemographic factors associated with COVID-19 vaccine hesitancy and reasons given by unvaccinated study participants against COVID-19 in Tanzania.

## MATERIALS AND METHODS

### Study design.

We conducted a mixed-method cross-sectional study with two components: health facilities and communities. The study provides quantitative and qualitative details. The qualitative part was mainly intended to explain the reasons for COVID-19 vaccine hesitancy, as a complement to the quantitative study. We report health-care provider, patient, and community knowledge, attitudes, and practices regarding COVID-19 vaccine hesitancy. Our study was conducted between March and September 2022.

### Study setting.

**Figure 1.**
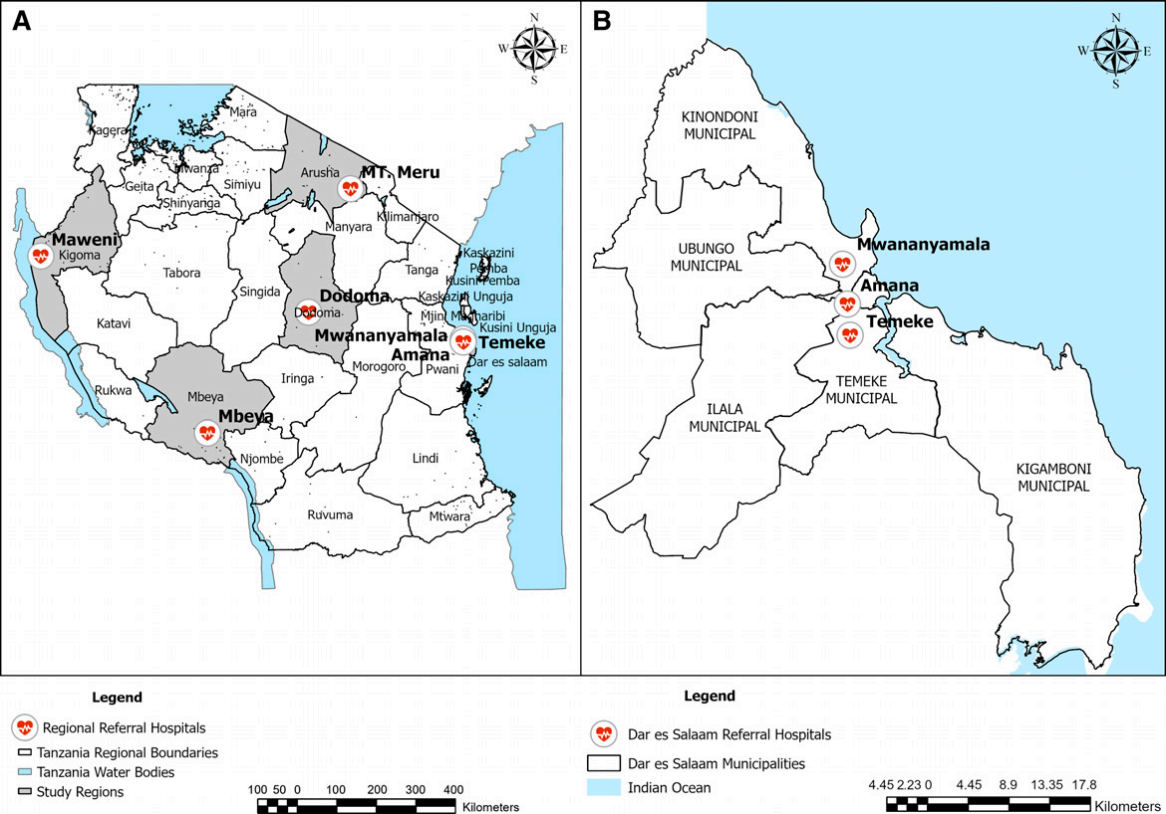
(**A**) The national set up of selected study regions and their respective referral hospitals in Tanzania. (**B**) An expanded version of selected study referral hospitals in Dar es Salaam region.

The health facility component was done in seven selected zonal and regional referral hospitals: the Mount Meru Regional Referral Hospital in Arusha (northern zone of Tanzania), the Maweni Regional Referral Hospital in Kigoma (northwestern zone of Tanzania), the Mbeya Zonal Referral Hospital in Mbeya (the southern highlands zone of Tanzania), the Dodoma Regional Referral Hospital in Dodoma (the central zone of Tanzania), and the Mwananyamala Regional Referral Hospital, Temeke Regional Referral Hospital, and Amana Regional Referral Hospital in Dar es Salaam (eastern zone of Tanzania). The community component of the study included three municipalities in Dar es Salaam: Kinondoni, Ilala, and Temeke. Dar es Salaam is a major city of Tanzania and the center of business, industry, and commerce, as detailed elsewhere.^32^ The map in Figure 1 depicts a selection of the study area in Tanzanian regions.

### Study procedure.

Individuals were enrolled in the survey if they met the following criteria: They were 18 years or older, they lived in the study site, and they were willing to complete the survey and sign an informed consent form. A structured questionnaire was used to collect quantitative data, capturing sociodemographic information; knowledge, attitudes, and practices related to COVID-19; and perceptions of the COVID-19 vaccine. The questionnaire was prepared in English and Swahili for both health facility and community surveys, and was loaded onto standardized data collection tablets, with the exception of the consent forms, which were filled out on paper.

The questionnaire was reviewed for validity and reliability by three senior researchers, and was modified based on their recommendations and suggestions. A pilot survey was then carried out on a small sample of the study population to test for acceptability and clarity, resulting in a concise and easily understandable questionnaire that took 30 to 45 minutes to complete. The final version of the questionnaire, approved by all investigators, consisted of the following three sections: 1) sociodemographic and other information; 2) knowledge, attitudes, and practices related to COVID-19; and 3) perceptions of COVID-19 antigen rapid diagnostic tests and the COVID-19 vaccine.

To record the sociodemographic information, participants were asked about their age, gender, marital status, level of education, and occupation (Table 1). The second section focused knowledge, attitudes, and practices regarding COVID-19. There were six questions each on attitudes and practices with regard to COVID-19, which were measured using a 5-point Likert scale (Always, Often, Sometimes, Rarely, Never). In addition, eight questions regarding knowledge of COVID-19—such as knowledge of symptoms, spread, and risk factors—were included and assessed with Yes or No answers. Knowledge scores greater than the mean indicated the participant had knowledge; scores less than the mean indicated a lack of knowledge.

**Table 1:** Study participant characteristics (*N* = 1,508)

Variable	Health facilities	Communities	Total
No. of participants surveyed and gives reasons, *n*	1,228	280	1,508
No. of unvaccinated study participants, *n* (%)	506 (41.2)	66 (23.6)	572 (37.9)
Participant information, *n* (%)			
Male	522 (42.5)	90 (32.1)	612 (40.6)
Female	706 (57.5)	190 (67.9)	896 (59.4)
Age category, years, *n* (%)			
18–40	582 (47.4)	213 (76.1)	795 (52.7)
> 40	646 (52.6)	67 (23.9)	713 (47.3)
Region, *n* (%)			
Arusha	79 (6.4)	0 (0.0)	79 (5.2)
Dar es Salaam	360 (29.3)	280 (100.0)	640 (42.4)
Dodoma	212 (17.3)	0 (0.0)	212 (14.1)
Kigoma	113 (9.2)	0 (0.0)	113 (7.5)
Mbeya	464 (37.8)	0 (0.0)	464 (30.8)
Marital status, *n* (%)			
Single	302 (24.6)	125 (44.6)	427 (28.3)
Married/cohabiting	680 (55.4)	129 (46.1)	809 (53.7)
Widow/widower	162 (13.2)	11 (3.9)	173 (11.5)
Divorced	84 (6.8)	15 (5.4)	99 (6.6)
Education levels, *n* (%)			
None	753 (61.3)	143 (51.1)	896 (59.4)
Primary school	261 (21.3)	104 (37.1)	365 (24.2)
Secondary and above	214 (17.4)	33 (11.8)	247 (16.4)
Employment status, *n* (%)			
Student	53 (4.3)	22 (7.9)	75 (4.9)
Formally employed	39 (3.2)	25 (8.9)	64 (4.2)
Unemployed	190 (15.5)	71 (25.4)	261 (17.3)
Self-employed	263 (21.4)	152 (54.3)	415 (27.5)
Business	95 (7.7)	10 (3.6)	105 (6.9)
Unknown	588 (47.9)	0 (0.0)	588 (39.9)

**Figure 2.**
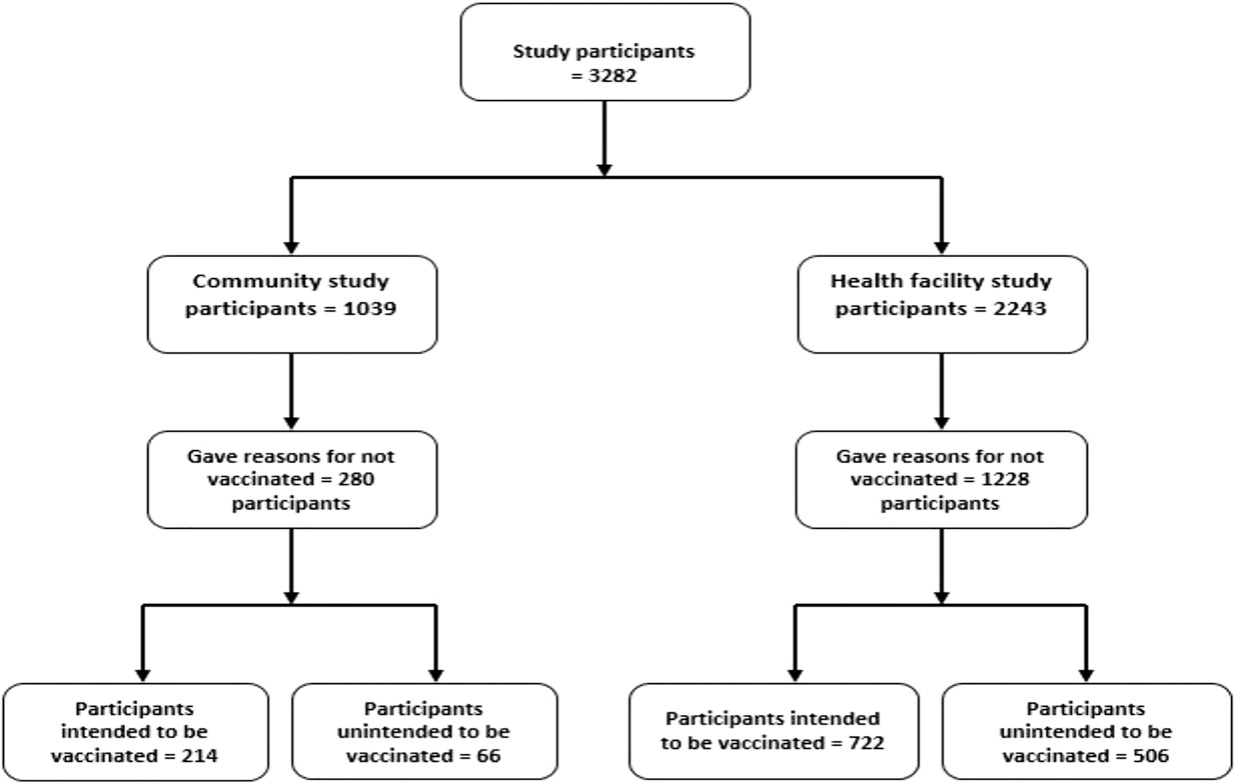
Schematic diagram of the number of participants sampled from cross-sectional survey conducted from health facility and community.

Six questions were asked that pertained to participants’ perceptions of the COVID-19 vaccine, including, “Have you received a COVID-19 vaccination?” The options Yes or No indicated their level of vaccine hesitancy. If respondents said, “No,” they had to explain why they were not vaccinated. Msuya et al.^24^ asked a similar question regarding the acquisition of the COVID-19 vaccine; respondents were able to select one of the following options: Yes, Will wait for some time before getting it, and Will not get the vaccine. Unfortunately, that study failed to extract participants’ reasons for those who chose to wait and those who chose not to get the vaccine. In our study, responses to “If not choosing to vaccinate, explain why” were examined thoroughly. Seven reasons emerged: fear of the safety and short-term side effects of the COVID-19 vaccine, lack of trust in or a low perceived benefit from the COVID-19 vaccine, belief in spiritual or religious views, belief in local remedies and other precautions or preventive measures, Belief in Fate/Lack of restrictions and compulsions to be vaccinated, insufficient knowledge about the importance and safety of the COVID-19 vaccine, and presence of multiple sources of information related to the COVID-19 vaccine. Participants who indicated they were undecided about whether to get the vaccine, they wanted to receive the vaccine, or they wanted to wait, for the reasons just listed, were categorized as intending to get the vaccine. Participants who responded, “I am afraid,” or “I don’t want to be vaccinated,” were classified as not intending to get the vaccine. Figure 2 presents a schematic diagram of the numbers of participants sampled from the cross-sectional survey conducted from health facilities and communities in Tanzania.

For the qualitative study, in-depth interviews (IDIs) were undertaken by the investigators. Thirty-four IDIs were conducted, of which 15 and 19 IDIs were done with health-care providers and patients, respectively (Supplemental Table 1). In Dar es Salaam, the IDIs were conducted at the Amana, Mwananyamala, and Temeke hospitals. Because patients and health-care providers comprised the study group, it was not feasible to conduct focus group discussions. Purposive sampling was used in both studies as a sampling strategy^33^ to select the participants for IDIs and included respondents from all groups. The sample size for these qualitative studies was determined by the point of saturation as a criterion for discontinuing data collection.^34^ The interviews were audio-recorded, and their responses were later transcribed and analyzed. For both studies, participants were assured their anonymity and confidentiality would be maintained, because all data provided by the participants were kept confidential.

### Outcome and exposure variables.

The primary outcome of COVID-19 vaccine hesitancy was dichotomized, with those who answered they had not been vaccinated but needed to be vaccinated characterized as “intend to get the vaccine” and those who answered they had no need for or were afraid of the vaccine characterized as “do not intend to get the vaccine.” The exposure variables included gender, age, education level, employment status, and marital status. We also included information about knowledge of symptoms, spread, and risks of COVID-19.

### Data analysis.

Descriptive statistics for categorical variables were summarized using frequency and percentage. Bivariate logistic regression was used to determine the association between the risk factors and outcome. Those predictors showing significance at a *P* value of less than 25% were considered for multiple logistic regression.

In addition to fitting the fully saturated model, we performed a stepwise regression, adding each variable successively to the model and balancing significant variables < 0.25 with parsimony.^35^ Multivariate logistic regression analysis was carried out to identify factors associated with vaccine hesitancy, expressed as an adjusted odds ratio (AOR) along with its respective 95% CI. The explanatory variables entered into the multivariate logistic regression include gender, age, marital status, education level, and knowledge about COVID-19. Religious variables were not included in data collection, and thus no analysis was performed on these variables, because religion is often a sensitive and controversial subject. Analyzing religious differences could potentially lead to misunderstandings or misrepresentations, and it can be challenging to maintain a neutral stance when discussing religious beliefs. To avoid potential conflicts or biases, we deliberately did not include these variables. The χ^2^ test was used to compare the proportion of reasons given by unvaccinated study participants by age, gender, and level of education.

All analyses were conducted using STATA (version 16; StataCorp, College Station, TX) and graphically analysis were done using R version 3.6.3 (R Foundation for statistical computing, Vienna, Austria, Available at: https://www.R-project.org/).^36^ We used the framework method to analyze the qualitative data, focusing on emerging themes, patterns, similarities, and differences.^37^ We also used open coding to label concepts, defining and developing categories based on properties and dimensions of participants’ descriptions.^38^ Codes were grouped into categories and during coding process, one researcher performed the initial coding, and then two researchers met to compare the codes versus the transcripts and reach a consensus on the final codes. We used both an inductive approach (ideas emanating from the data itself) and a deductive approach (theoretical understanding, literature review, and researcher’s experience) for data analysis. All qualitative analyses were performed using NVIVO (version 12; QSR International, Boston, MA).

## RESULTS

### Sociodemographic characteristics of the study participants.

A total of 1,508 people were approached and agreed to participate in the survey, which was conducted at various health facilities and communities, and provided reasons for not being vaccinated. Of the participants, 572 (38.0%) from both groups (health facilities and communities) did not intend to be vaccinated against COVID-19. In both surveys, women outnumbered men (*n *= 896, 59.4%). Youths and middle-aged adults between 18 and 40 years were more likely to be found in communities (*n *= 213, 76.1%), whereas those older than 40 years were more likely to be found in health facilities (*n *= 646, 52.6%). In comparison to other regions, Dar es Salaam had the greatest number of surveyed participants (*n *= 640, 42.4%). For both studies, a total of 809 study participants (53.7%) were married or cohabiting. The majority of surveyed participants in the health facility (*n *= 753, 61.3%) and community survey (*n *= 143, 51.1%) had not completed primary school. A total of 415 study participants were self-employed, and fewer (*n *= 64, 4.2%), were formally employed. The baseline characteristics of the study participants are shown in Table 1.

### Reasons for not be vaccinated against COVID-19.

**Figure 3.**
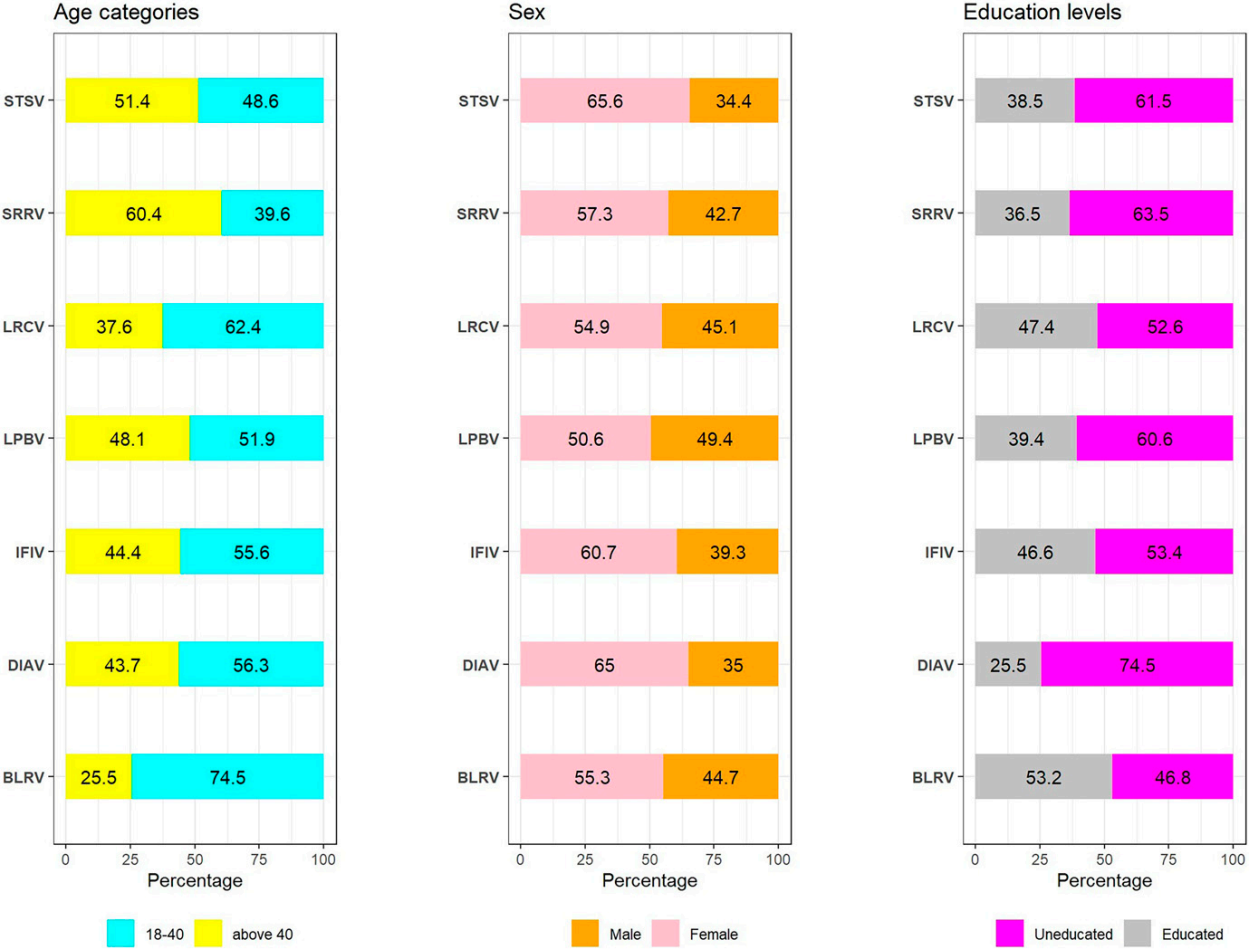
Reasons for not be vaccinated against COVID-19 disease among study participants based on sociodemographic characteristics (*N* = 1,508). The abbreviations used for y-axis are as follows: STSV = Fear of the safety and short-term side effects of the COVID-19 vaccine; SRRV = Belief in spiritual or religious views, LRCV - Belief in Fate/Lack of restrictions and compulsions to be vaccinated; LPBV = Lack of trust or low perceived benefit in the COVID-19 vaccine; IFIV = Insufficient knowledge on importance and safety of the COVID-19 vaccine; DIAV = Difficulty accessing or having irregular access to COVID-19 vaccination centers; BLRV = Belief in local remedies and other precautions or preventive measures.

To shed more light on vaccine hesitancy issues, we investigated the reasons for not be vaccinated against COVID-19 among a representative sample of the study population by sociodemographic characteristic (age, gender, and level of education) in five Tanzanian regions (Arusha, Dar es Salaam, Dodoma, Kigoma, and Mbeya). We discovered a significant difference in the reasons given for those who intended to get the vaccine and those who did not intend to get the vaccine, based on their age, gender, and level of education. We categorized education as “educated” for those who completed primary and secondary education, and “uneducated” for those who had no formal education. Figure 3 illustrates the reasons for not being vaccinated against COVID-19, categorized by sociodemographic characteristics (age, gender, and level of education).

#### Fear of the safety and short-term side effects of the COVID-19 vaccine.

With regard to age, adult participants older than 40 years (51.4%) were significantly (χ^2^, *P* < 0.001) unvaccinated because of their fear of the safety and short-term side effects of the COVID-19 vaccine compared with unvaccinated youth and middle-aged adults between 18 and 40 years (48.6%). Women were not vaccinated (65.5%) more than men (34.4%) because of concerns about the vaccine’s short-term side effects (χ^2^, *P* < 0.001). When compared with educated respondents (38.5%), a significantly greater proportion of uneducated study participants (61.5%; χ^2^, *P* < 0.001) did not intend to be vaccinated because they were afraid of the short-term side effects of vaccine (Figure 3).

In the qualitative study, we also discovered that vaccine hesitancy was facilitated by concerns of the COVID-19 vaccine short-term side effects. Participants hinted at worrying about the vaccine’s side effects, which they claimed were very uncomfortable and, in some cases, life-threatening. They claimed the vaccine’s side effects prevented them from acquiring it. The following are the COVID-19 vaccine short-term side effects mentioned by the study respondents and their responses:

1. COVID-19 vaccine causes paralysis.
  Really, its safety is not well trusted because already people have seen it having side effects. Because there is someone got vaccine, and has been affected, others have paralyzed. I am not aware of which criteria does the vaccine need to suit you. So you find people saying, “Why should I get vaccinated? It is better to just live. If I will die, that’s over.” [IDI Mbeya, female participant no. 03, 25 years]
  [R]eally, other like us, it like the dates [days to live] are over. We should just [die], because [the vaccine] can affect you directly. For example, one of my neighbor, got stroke and started to paralyze. He/she said, “After getting the vaccine, I got stroke. I didn’t have another problem.” [IDI Kigoma, male participant no. 03, 60 years]
2. COVID-19 vaccine causes erectile dysfunction and inability to conceive. One of the health-care providers reported that people do not want to be vaccinated because they have heard it can interfere with male erectile function (impotence) and female infertility.
  There are those who accept and others scorn the vaccine. Not all [people] can accept [the vaccine]. Everyone has the way to receive information about the vaccine… . As for them [talking] about the vaccine, they say it reduce male sexual ability, and inability to conceive … . The reception [of the information] differs from one person to another. [IDI Mbeya, health provider participant no. 03, 41 years]
  There were people who said that [the vaccine] is safe; [it has] no problem. But also there were people who have been brainwashed that there is no safety. There are many [side effects] which were talked about, including man sexual ability (you will be functionless) [and] women will not conceive because [the vaccine is] going to destroy ovaries. So all two sides do exist. Those who perceive that it is safe, there is no any problem. But also, [there are] those who said, “Hmm, there is problem there.” Until now, they believe that there is problem within the vaccine. [IDI Mbeya, health provider participant no. 02, 41 years]
3. Threat from other people that COVID-19 vaccine causes blood clotting and death. Some participants also revealed what their community members say about the COVID-19 vaccine in relation to blood clotting and death.
  [P]eople are so eager to be vaccinated, but they fear the way people appear. A person can say to you that, if you vaccinate and start walking, the blood clot will occur. So people fail to vaccinate. [Interviewer: Is there a person who has ever been vaccinated and got a blood clot?] Yes, we just heard there in the Democratic Republic of Congo! I heard they vaccinated and the blood clotted. [IDI Kigoma, female participant no. 01, 39 years]
  Initially, people were threatened about the [vaccine] injections—that if you get injected, it will kill you. But as the days go on, people get understanding of the meaning of the vaccines. But, at the beginning, people got worried so much. “They may cause blood to clot, and have side effects.” But now, as the time goes on, education is increasingly delivered. A person has no worry. [IDI Dodoma, female participant no. 02, 45 years]
  Other say that they heard from community that when you get vaccinated, the blood clot. [IDI Dar es Salaam, male participant no. 01, 29 years]
4. Belief that COVID-19 vaccine causes critical illness and death. It was also noted from the participants that the vaccines cause people to become critically ill when they get vaccinated, which sometimes leads to death. For that reason, some people didn’t accept the vaccine and remain unvaccinated.
  People are becoming critically ill [because of the vaccine]. Example is from where I was working. There was a gentleman, was a really rich—the Don [extremely rich]! But he died because he got vaccinated for the COVID-19 disease. Soon after being vaccinated, he started getting sick. Finally, he died. So from that point, I got totally despaired. I found myself being far away from the COVID-19 vaccine. [IDI Mbeya, female participant no. 03, 25 years]
  In the community I live, I think there are mixed feelings. There are people who accept it and they have accepted to be vaccinated. But until now, there are other people who are still worried, saying the vaccine has side effects. People are dying. You see? Eh! Therefore, until, now there are people who have not yet accepted it and they have not accepted even to be vaccinated. [IDI Mbeya, health provider participant no. 01, 44 years]

#### Lack of trust in or low perceived benefit from the COVID -19 vaccine.

The significantly greater proportion of uneducated unvaccinated study participants (60.6%; χ^2^, *P* = 0.027) than educated (39.4%) study participants mentioned a lack of trust or low perceived benefit of vaccination (Figure 3). In the qualitative study, the following aspects and responses to them were noted in this category:

1. Procedure to get vaccinated. The procedure included the need to sign a consent form. To some, this requirement implied the vaccine might not be safe. In addition, participants stated that giving consent is the same as giving your life away, and this was seen as a risk:
  Concerning the vaccine, we are rejecting it because it contains numerous ingredients. [And] it threatened us that when you register for vaccination, it has two: the same vaccine you swear to and then you get vaccinated. That you should not complain to anyone about whatever happened to you. Now that vaccine worried us so much, which is why people don’t want it. Who will you complain to if you are sworn like that and die? Did you not register yourself? That’s exactly the point! [IDI Dar es Salaam, male participant no. 02, 69 years]
2. Disease outcome to that the vaccinated and unvaccinated are the same:
  They don’t believe the vaccine. [Interviewer: Why don’t they believe it?] They don’t believe because, my view is that, the one who has vaccinated and the one hasn’t, are the same, as they have not seen the one who has been vaccinated and got infected [with COVID-19], has got healed. You know, people we do expect to see something . . . . [Interviewer: So the majority of those vaccinated for COVID-19, if they get infected, they can’t heal?] They can’t get heal, and they don’t say if they have got vaccinated. [IDI Kigoma, female participant no. 03, 60 years]

#### Belief in spiritual or religious views.

The older study participants who believe in spiritual or religious views were significantly more likely to be unvaccinated (60.4%; χ^2^, *P* = 0.001) than unvaccinated youth and middle-aged adults (39%). On the other hand, when compared with educated participants (36.5%), approximately 63.5% of uneducated study participants (χ^2^, *P* = 0.08) who were unvaccinated believe in spiritual and religious views (Figure 3).

In the qualitative study, it was reported that believers of particular religious sects do oppose the vaccine because they believe the vaccine cannot prevent COVID-19. They only believe that God can prevent COVID-19 from happening:

[S]some of them have their own belief that these vaccines are not for us; the vaccine is for [type of religion sect]. So those who believe that, they do exist, and if you tell [them] about the vaccine, [they don’t] understand you. In addition, [they say] the vaccine is for [type of religion sect] only. [Interviewer: Have you heard them saying that exactly?] I have exactly heard that [in] *daladala* [urban commuter bus] when coming back [home]. [T]here were many stories in transport facilities. [IDI Dar es Salaam, health provider participant no. 3, 32 years]

About the vaccine and its safety, to where I live, many people really do not accept to take the vaccine, and those who accept the vaccine, also do exist. And many of those who do not accept the vaccine, are those who praying [type of religious sect]. Those with the word of Lord, they don’t believe that the COVID-19 vaccine can prevent, but they believe God can prevent it from happening, and end [the COVID-19 pandemic]. [IDI Mbeya, female participant no. 03, 25 years]

#### Belief in local remedies and other precautions or preventive measures.

The high proportion of unvaccinated youths and middle-aged people choose to be unvaccinated because they believe in local remedies (74.5%; χ^2^, *P* = 0.04) (Figure 3). In the qualitative study, some participants reported hearing from a political leader that COVID-19 can be cured with local remedies:

Our [title deleted] told us that the treatment [for COVID-19] is cured even by pawpaw leaves and bathing with local remedies. You take pawpaw and boil [it with water]. You bath and drink. That’s all. [IDI Dar es Salaam, male participant no. 02, 69 years]

Furthermore, other participants stated that some community members did not believe in the vaccine and instead chose other precautions or preventive measures such as social distancing, wearing masks, and handwashing rather than vaccination:

My community was not yet to believe in vaccination, because they saw in the first period of the disease outbreak, in which many people . . . abroad got vaccinated, we saw them were not healed. And that other people, despite being [vaccinated], . . . were infected by the disease. Thus why they took the first precaution [social distancing, wearing masks, and handwashing]. [IDI Arusha, male participant no. 03, 27 years]

#### Belief in Fate/Lack of restrictions and compulsions to be vaccinated.

The greater proportion of youths and middle-aged people were unvaccinated because of a lack of restrictions and compulsions to be vaccinated (62.4%; χ^2^, *P* = 0.04) than older unvaccinated participants (37.6%) (Figure 3). In the qualitative study, it was noted that the majority of people in the community were against the vaccine. They believe that regardless of whether you get vaccinated, if your “time is up,” you will die. So for that reason, they conclude there is no need to get vaccinated:

[F]irstly, in a huge percent of people, if you tell a person to vaccinate, he/she tell you that [the vaccine] is meaningless. [Interviewer: Why are they saying it is meaningless?] Because whether you vaccinate or not, you must die. So, you see now there is no reason to get vaccinated. [Those are] majority opinions from where we live. Because after these seminars, I have walked around to mobilize about the vaccine, but later I discovered that [they are] saying, “Ah, we don’t want vaccine issues.” [IDI Kigoma, male participant no. 03, 60 years]

#### Insufficient knowledge on the importance and safety of the COVID-19 vaccine.

A few of the participants had views that the vaccine was introduced quickly and the community was not well informed about the importance and safety of vaccines:

I have not vaccinated and I will not, because I have not been given enough information on the importance of the COVID-19 vaccines. The vaccines were rapidly introduced and the community has not been well informed. [IDI Arusha, male participant no. 04, 20 years]

Because I . . . never heard [about the vaccine, which is] why there was the question which I had asked before, that, “For what percent does it help?” Because I have never heard its safety and I have never made follow-up of it. [IDI Kigoma, female participant no. 04, 19 years]

#### Presence of multiple sources of information related to the COVID-19 vaccine.

Further reason for hesitation was a result of the presence of various sources of COVID-19 vaccine information, some of which contradicted the COVID-19 vaccine information provided by the government:

After education being provided, others had understood. Those who didn’t understand, they shall not understand ’til in the grave; but, it is due to contradiction of the government statements. That is because the main spokesperson might be the head of state or the minister responsible with the ministry. But every person is speaking, “This causes blood clotting.” “If you do this, you cause male sexual inability.” “Women will not conceive!” Just imagine for a person who have no any understanding, has no any education, what will he/she do? [IDI Dodoma, male participant no. 03, 58 years]

There are people who emerged out from social media, not only in Tanzania, but even abroad. They claim that, “Ooh! The person has got vaccinated; he/she got blood clot problem in blood platelets.” [Another] one said that it causes inability to conceive. But all they didn’t come with scientific evidence. [IDI Dar es Salaam, health provider participant no. 01, 26 years]

### Correlate of vaccine hesitancy against COVID-19 disease.

In the bivariate logistic regression analysis, age, gender, marital status, level of education, employment status, and general knowledge of COVID-19 were identified as statistically significant factors for not intending to receive COVID-19 vaccination. After controlling for other risk factors, female participants had a 51% greater estimate of no intent to be vaccinated than men (AOR, 1.51; 95% CI, 1.19–1.90; *P* = 0.001). In terms of age, it is estimated that participants older than 40 years had a 28% greater risk of not intending to be vaccinated compared with participants between 18 and 40 years (AOR, 1.28; 95% CI, 1.01–1.61; *P* = 0.04). Furthermore, in the multivariate analysis, participants who knew about COVID-19 symptoms had a 21% less risk of not intending to be vaccinated (AOR, 0.79; 95% CI, 0.61–1.02; *P* = 0.07). Also, there was a negative association between not intending to be vaccinated and knowing about the spread of COVID-19 (AOR, 0.62; 95% CI, 0.45–0.85; *P* = 0.003) (Table 2).

**Table 2:** The predictors and factors associated with COVID-19 vaccine hesitancy in selected community and health facilities

Variable	Will be vaccinated, *n *(%)	Will not be vaccinated, *n *(%)	Bivariate logistic regression			Multivariate logistic regression		
			OR	95% CI	*P *value	AOR	95% CI	*P* value
No. of participants who gave reasons	936	572	–	–	–	–	–	–
Gender								
Male	415 (44.3)	197 (34.4)	Ref.	Ref.	Ref.	Ref.	Ref.	Ref.
Female	521 (55.6)	375 (65.6)	1.52	1.22–1.88	< 0.001	1.51	1.19–1.90	0.001
Age category, years								
18–40	517 (55.2)	278 (48.6)	Ref.	Ref.	Ref.	Ref.	Ref.	Ref.
> 40	419 (44.8)	294 (51.4)	1.31	1.05–1.61	0.01	1.01	0.79–1.29	0.91
Region								
Arusha	64 (6.8)	15 (2.6)	Ref.	Ref.	Ref.	Ref.	Ref.	Ref.
Dar es Salaam	448 (47.9)	192 (33.6)	1.83	1.02–3.29	0.04	1.51	0.81–2.77	0.19
Dodoma	56 (5.9)	156 (27.3)	11.88	6.27–22.54	< 0.001	10.62	5.53–20.39	< 0.001
Kigoma	76 (8.1)	37 (6.5)	2.08	1.05–4.12	0.04	1.59	0.77–3.26	0.21
Mbeya	292 (31.2)	172 (30.1)	2.51	1.39–4.55	0.002	2.04	1.09–3.79	0.02
Marital status								
Single	269 (28.7)	158 (27.6)	Ref.	Ref.	Ref.	–	–	–
Married/cohabiting	507 (54.2)	302 (52.8)	1.01	0.79–1.29	0.671	–	–	–
Widow/widower	97 (10.4)	76 (13.3)	1.33	0.93–1.91	0.022	–	–	–
Divorced	63 (6.7)	36 (6.3)	0.97	0.62–1.53	0.119	–	–	–
Education level								
None	544 (58.1)	352 (61.5)	Ref.	Ref.	Ref.	Ref.	Ref.	Ref.
Primary school	226 (24.2)	139 (24.3)	0.95	0.74–1.22	0.69	1.25	0.94–1.69	0.13
Secondary and above	166 (17.7)	81 (14.2)	0.75	0.56–1.02	0.05	1	0.69–1.45	0.99
Employment status								
Student	42 (4.5)	33 (5.8)	Ref.	Ref.	Ref.	–	–	–
Formally employed	38 (4.1)	26 (4.6)	0.87	0.44–1.71	0.69	–	–	–
Unemployed	134 (14.3)	127 (22.2)	1.2	0.72–2.02	0.48	–	–	–
Self-employed	270 (28.9)	145 (25.4)	0.68	0.42–1.13	0.14	–	–	–
Business person	78 (8.3)	27 (4.7)	0.44	0.23–0.83	0.01	–	–	–
Unknown	374 (39.9)	214 (37.4)	–	–	–			
Do you know the symptoms of COVID-19?								
Don’t know symptoms	342 (36.5)	245 (42.8)	Ref.	Ref.	Ref.	Ref.	Ref.	Ref.
Know symptoms	594 (63.5)	327 (57.2)	0.77	0.62–0.95	0.02	0.79	0.61–1.02	0.07
Do you know how COVID-19 spread?								
Don’t know	686 (73.3)	450 (78.7)	Ref.	Ref.	Ref.	Ref.	Ref.	Ref.
Know	250 (26.7)	122 (21.3)	0.74	0.58–0.95	0.02	0.62	0.45–0.85	0.003
Do you know the risks of contracting COVID-19?								
Don’t know	675 (72.1)	406 (71.0)	Ref.	Ref.	Ref.	Ref.	Ref.	Ref.
Know	261 (27.9)	166 (29.0)	1.05	0.84–1.33	0.64	1.25	0.93–1.68	0.13
Do you think COVID-19 can be prevented?								
Yes	689 (73.6)	412 (72.0)	Ref.	Ref.	Ref.	Ref.	Ref.	Ref.
No	247 (26.4)	160 (28.0)	1.11	0.86–1.37	0.5	0.98	0.74–1.29	0.88
Do you think COVID-19 is a serious disease?								
Yes	821 (87.7)	504 (88.1)	Ref.	Ref.	Ref.	Ref.	Ref.	Ref.
No	115 (12.3)	68 (11.9)	0.96	0.69–1.33	0.81	1.16	0.81–1.65	0.43

AOR = adjusted odds ration; OR = odds ratio.

## DISCUSSION

Although several studies are being carried out in different parts of the world regarding COVID-19 vaccine hesitancy, there are limited studies in Tanzania at the moment. In our study, we aimed to provide new insights into the sociodemographic factors hindering COVID-19 vaccination in Tanzania. Our results revealed a greater rate of vaccine hesitancy (37.9%), with the most commonly mentioned reasons being fear of the safety and short-term side effects of the vaccine. This hesitancy is slightly greater than that of a study from Pakistan (35.6%),^39^ with the reasons given consistent with our findings, and greater than that of the United States (20%),^40^ with the reasons given being the novelty of the vaccine and concerns about its safety.

In contrast, vaccine hesitancy in our study population was less than a study from Australia (41%),^41^ where hesitancy was a result of those with more populist views and greater levels of religiosity, and from Saudi Arabia (55.3%),^42^ where hesitancy was a result of concerns about vaccine side effects. Some possible explanations for this include a lack of trust in medical organizations and vaccine providers, as well as the belief that a vaccine could not save someone from COVID-19, and there was no need to get vaccinated if precautionary measures were taken.

Women were less likely to be vaccinated than men. Approximately 65.6% of women reported they did not intend to get vaccinated because of concerns about the vaccine’s short-term side effects. These findings are consistent with vaccine surveys in Ethiopia,^43^ where female participants were concerned about the vaccine’s efficacy; as well as in China,^44^ where women were concerned about vaccine safety; and in the United Kingdom,^45^ where women were concerned about future unforeseen side effects of the COVID-19 vaccine. Other studies^7^^,^^46^ have identified additional reasons for vaccine hesitancy among women, including beliefs that the vaccine may not protect their health adequately, which makes women less willing to get vaccinated than men. Furthermore, research suggests that women are more cautious and take longer to evaluate risk than men, as demonstrated by studies conducted by Szilagyi et al.^47^ and Inglehart and Noris,^48^ who assessed personality traits related to risk perception. Therefore, further research is needed to investigate the influence of factors such as risk perception, access to health care and health literacy, attitudes and beliefs, differential COVID-19 vulnerability, and comorbidity burden based on gender, as well as on factors such as parenthood and family structure. Although we collected data on gender, according to the scope of our study, we did not collect information regarding whether women feel empowered to vaccinate.

Our findings show that 51.4% of adults study participants older than 40 years did not intend to be vaccinated because of fear of short-term side effects of the COVID-19 vaccine, compared participants between 18 and 40 years. This percentage is greater than a study conducted in the United States, which discovered that nearly 31.1% of adult participants did not intend to receive a COVID-19 vaccine^49^ because of concerns about vaccine safety and effectiveness.

According to the findings of our qualitative study, the reasons for vaccine hesitancy in Tanzania were the possibility that the COVID-19 vaccine could cause paralysis. This finding contradicts other studies^50^^,^^51^ that concluded that, when compared with other viral vaccines in the pharmacovigilance database that cause paralysis such as facial paralysis, messenger RNA COVID-19 vaccines did not show any sign of causing facial paralysis. If it does exist, the risk is likely to be very low, as with other viral vaccines. With regard to COVID-19 vaccine causing erectile dysfunction and an inability to conceive, there is no solid evidence connecting the COVID-19 vaccine to sperm quality impairment,^52^ nor concerns about infertility and vaccine safety while pregnant or breastfeeding. Therefore, it would be beneficial for health-care professionals to dispel myths about COVID-19 vaccines and reproductive health, given prior research indicating that expert correction of health misinformation can reduce misinformed beliefs.^53^

Furthermore, our research shows that older people did not intend to be vaccinated because of their spiritual or religious views. This finding is consistent with another study^54^ that showed how religion influences people’s willingness to acquire the COVID-19 vaccine. The possible explanation for this is that their religion might prevent them from getting vaccinated, or study participants may believe that the COVID-19 vaccine contains ingredients prohibited by their religion. In terms of religious beliefs, participants who were hesitant or refused to get vaccinated cited religious leaders’ advice as one of the main reasons for not getting vaccinated. This finding is consistent with previous reports^55^ that demonstrated the critical role of religious leaders in vaccination program uptake. Because the possible causes could stem from religious doctrine, there is a need for discussions among religious leaders, public health authorities, and medical experts to respond and clarify concerns among religious communities.

In our study, 74.5% of adults between the ages of 18 and 40 did not intend to be vaccinated because they believe in local remedies and other precautions or preventive measures. This finding is consistent with research conducted in Africa and Asia, where traditional remedies are regarded as a major source of illness treatment^56^ and, in some cases, as first-line treatments.^57^ Some possible explanation for this is that some home remedies may be able to treat mild COVID-19-like symptoms, which could lead to a greater reliance on traditional remedies rather than the COVID-19 vaccine. Lower educational attainment has been noted to be an ongoing social vulnerability for vaccine hesitancy in both Tanzania and the United States,^58^ along with other studies^42^^,^^59^^,^^60^ that discovered associations between low education and vaccine hesitancy. Some studies have found the opposite^61^^–^^63^ showing that people with less education are more likely to receive vaccinations. Other studies^41^^,^^64^ have also linked education level to vaccine reluctance, suggesting that this may be because people understand the threat and the vaccine at different levels. In some instances, participants reported that education about the vaccine, its side effects, and its efficacy, coupled with positive feedback from those who had been vaccinated, would encourage them to get vaccinated. The main source of information for participants was the ministry website, as demonstrated in another study.^65^ Because education has been found to be a significant predictor of vaccine hesitancy,^66^^–^^68^ this may also have been related to health literacy, which raises the need for additional research to substantiate this theory.

According to Tanzanian literature, parental concerns about insufficient vaccination knowledge, lack of information about vaccines and their benefits, and a lack of access to vaccines are factors in vaccine hesitancy for children who are scheduled for vaccination.^69^ Because of the difficulties and inconsistency of access, mothers must travel to distant places to visit vaccination sites. When patients arrive, they sometimes find the vaccine are out of stock, causing health-care providers to ask them to return to the clinic. Concerning lack of trust or perceived value of childhood vaccines, it is noted that the caregivers of children who miss a vaccination have low vaccine confidence, as do those who forget to vaccinate their child and those who believe vaccines are unnecessary. These parents/caregivers discussed their concerns regarding their children’s pain and minor adverse effects from vaccination (such as crying and the development of abscesses), as well as their opinion of the vaccine’s inefficiency. Furthermore, it has been demonstrated that the majority of these caregivers were reported to have had a negative experience with a previous vaccination and that their friends or family advised them not to receive the vaccine.^70^ In terms of vaccination hesitancy, only 57% of Tanzania’s eligible 18-month-old children received the measles-rubella vaccine in 2015, falling short of the WHO-recommended coverage rate of 95%.^71^ In addition, hesitancy to accept services for antiretroviral therapy and HIV testing is also great in Tanzania, as reported previously.^72^^,^^73^

With regard to a potential link between vaccine hesitancy in general and vaccine hesitancy related specifically to COVID-19, based on available research and observations, there appears to be a connection between vaccine hesitancy overall and a hesitancy toward getting a COVID-19 vaccine. It is crucial to highlight, however, that vaccination hesitancy is a complicated issue driven by a variety of individual, societal, and cultural variables. Understanding these issues is therefore critical for establishing effective communication and intervention techniques to increase vaccination adoption and public health interventions during the current COVID-19 pandemic.

We acknowledge that our study’s findings have both strengths and limitations. Our findings must be interpreted in light of their limitations. Our study’s cross-sectional design limits our ability to infer causal relationships. Furthermore, the survey represents respondents’ reasons and beliefs at a single point in time, which are subject to change. Also, other studies show an association between vaccine hesitancy and comorbidities, which may be potential confounders that are not addressed adequately in this work.

## CONCLUSION

The results of our study reveal an alarming situation for policymakers in Tanzania, as approximately 37.9% of study participants were hesitant to acquire a COVID-19 vaccination. To ensure greater acceptance of the COVID-19 vaccine by the general public, the government should conduct further evaluations to verify these findings and understand more fully the different demographic groups that are vaccine hesitant. Such an understanding would also assist governments and other organizations worldwide in their efforts to prepare for an upcoming pandemic—the type, name, and scope of which we cannot foresee—and in avoiding repeating the difficult lessons learned from the COVID-19 pandemic. By doing this, we can gain knowledge about how to support and enhance vaccine coverage among the general population as well as set specific, targetable innovations and interventions that can be used as a measure for the future—from crisis support to pandemic preparedness.

## Supplemental Materials


Supplemental materials
