# Supplementary material for: Understanding Sociodemographic Factors and Reasons Associated with COVID-19 Vaccination Hesitance among Adults in Tanzania: A Mixed-Method Approach
Source: Am J Trop Med Hyg. 2023 Sep 11;109(4):895–907. doi: 10.4269/ajtmh.23-0229 (PMC10551072; doi:10.4269/ajtmh.23-0229)
Supplement: Supplementary file 1 [file tpmd230229.SD1.pdf]

**Annex 1**

**Supplementary Table 1. Description of qualitative data distribution (34 IDI interviews)**

| | Regions | | | | |
|---|---|---|---|---|---|
| **Category** | **Mbeya** | **Dar es Salaam** | **Arusha** | **Kigoma** | **Dodoma** |
| **Health care providers** | **3** | **3** | **3** | **3** | **3** |
| **Patients (None COVID)** | **4** | **4** | **4** | **4** | **3** |
| *Male* | *2* | *2* | *2* | *1* | *1* |
| *Female* | *2* | *2* | *2* | *3* | *2* |
| **Total** | **7** | **7** | **7** | **7** | **6** |
